# Progress in Psychiatry

**Published:** 1901-10-19

**Authors:** 


### PROGRESS IN PSYCHIATRY.

**Puerperal Insanity, its Ætiology and Classification.**—This term is somewhat artificially restricted to insanity occurring within six weeks of childbirth, *i.e.* during the puerperal state in women. It is thus distinguished from the insanity of pregnancy, and from the insanity of lactation, the latter including those mental disorders occurring after six weeks of childbirth. In a recent publication Dr. Arthur C. Jelly,[1] of Boston, gives an account of puerperal insanity drawn from the medical records of the city of Boston for a period of eighteen years (1872-1890), and including 250 cases of puerperal mental disorder. Of these 250 cases the disorder was sufficiently serious in 200 cases to constitute insanity. From an ætiological point of view two groups of puerperal insanity are recognised. (*a*) In the first group the causes are septic infection (lacerations and various forms of metris and vulvitis) and general exhaustion, complicated in some cases with simple mastitis and auto-intoxication from the bowel. In (*b*), the second group, a predisposition to insanity or mental disorder already exists, and the ordinary stresses and sufferings of the puerperium are sufficient to cause the predisposition to manifest itself in actual insanity. The relative frequency of puerperal insanity in confinements has been variously stated: it is said to occur in 1 in 386 (Hansen), 1 out of every 460 deliveries (Clouston), and 1 in 700 (Menzies, Jones).[2] The insanities of pregnancy and of lactation, according to the statistics of Dr. Jelly, claim each about one-half (lactation rather more and pregnancy rather less) of the number of victims of puerperal insanity. Before the advent of general aseptic methods in obstetric practice puerperal insanity was much more frequent than it is to-day, but during the last three decades cases of puerperal insanity of septic origin have shown a tendency to decline steadily. Thus, in the Philadelphia Maternity Hospital 99 cases of puerperal insanity of septic origin were admitted in the period 1867-1877, while in the period 1887-1897 the number had fallen to 20, "the latter period being," says Dr. Jelly, "marked by the introduction of stricter antiseptic measures in obstetric practice in Philadelphia." In Boston there has been a decrease in the ratio of puerperal insanity to total labour cases, as shown in the following table:—

| Years. | Ratio of puerperal insanity to total labour cases. |
|---|---|
| 1872-1880... ... ... | ... 6·4 per cent. |
| 1880-1890... ... ... | ... 4 6 „ |
| 1890-1900... ... ... | ... 3·7 „ |

Hoche, of Hamburg, in 2,454 insane women found 98 cases of puerperal insanity proper, 89 of lactational insanity, and 24 cases of the insanity of pregnancy. As regards predisposition, Dr. Jelly finds that one-third of all his cases of puerperal insanity showed a history of insanity, epilepsy, or other cerebral disorder. Hoppe gives a slightly lower figure, while Idanoff gives 56 per cent. Alcoholism was also found to play an important part in some instances, either in the form of an acquired habit of intemperance or in the form of an hereditary taint. In 20 of Dr. Jelly's patients some distinct form of mental alienation or insanity had existed before pregnancy; thus one was a general paralytic, three were epileptic, three were imbecile, three were subjects of *dementia præcox*, five had delusional insanity, and five were suffering from secondary dementia. In a few instances it was found that during or immediately after the shock and pains of childbirth the patient developed sudden transitory delirious excitement or confusion, which, however, rapidly passed away when delivery had taken place. In a few cases the administration of ether or chloroform has provoked similar disorder. In all such cases a predisposition to epilepsy or profound hysteria is said to have existed (Kraepelin). The possibility of infanticide under such conditions should always be borne in mind, or of other, but less frequent, dangerous impulses. The importance of the moral factor as a predisposing cause has been shown clearly in the fact that puerperal insanity is found to occur about twice as frequently (or, according to Dr. Jones,[3] four times as frequently) after illegitimate as compared with legitimate births. Moreover the special racial proclivity of Jewesses to puerperal mental disturbance (in comparison with the native races of Austria, Russia, and the United States) has been pointed out by several observers cited by Dr. Jelly[4] in his paper. Puerperal insanity from septic or toxic infection may be looked upon as the main and most characteristic form of puerperal insanity, having an especial symptomatology of its own. Indeed in many points, as regards its causation, onset, and course, it resembles certain febrile deliria of micro-parasitic origin, and indeed is so included and classified by Lloyd Andriezen in a group with acute delirium, the grave delirium of influenza, scarlet fever, and acute cerebral meningitis.[5] As in other varieties of febrile insanity it is the unstable or neuropathic brain that yields readily to the toxic agencies and the pyrexia, just as in the case of children those of an unstable cerebral organisation readily develop delirium and hallucinatory mental confusion, whereas the more robust and healthy do not. In the few cases of puerperal insanity with fever and delirium where bacteriological examination has been made, with all needful precautions, of the uterine contents, the presence of pyogenic streptococci has been found either directly in the contents or in cultures inoculated with them, and in some fatal cases the pathways of infection (secondary suppurations and thrombi) have been traced in the body of the uterus, in the tubes and ovaries, in the pelvic peritoneum, and in the large veins of the pelvis. It is probable that in cases of severe perinæal ruptures and lacerations the tissues may get infected by the bacillus coli communis and other micro-organisms evacuated from the bowels, the bruised and damaged tissues offering but little resistance to the growth and invasion of such organisms. Infection from the mammæ is rare during the first week after childbirth, but the breasts should be carefully inspected for fissures, soreness and abrasions, mastitis and abscesses as sources of irritation and infection second only in importance to the uterus and genital tract. The organisms found within the tissues in fatal cases of puerperal febrile delirium have been cocci, especially streptococci and staphylococci in the uterine and pelvic cellular tissues and lymphatics, and abundantly in the purulent or sero-purulent fluid in the peritoneal cavity or in thrombi in the pelvic blood-vessels. The streptococcus pyogenes has been most often noted, but the staphylococcus pyogenes (aureus and albus) have also been found (Heiberg,[6] Klein,[7] Watson Cheyne,[8] and others). Besides hallucinatory delirium and acute mental confusion, there are various other diseases following the puerperium that are traceable to toxicæmia or infection, viz. a polyneuritis supervening within a few days of childbirth (Möbius, Bernhardt, Eulenberg [9]). In some cases extensive loss of blood during and after parturition (from placenta prævia, partial placental detachment, uterine inertia, and difficult and instrumental labours) may give rise to mental confusion and exhaustion with more or less afebrile delirium, a condition comparable to a similar mental disturbance which may arise from profound hæmorrhage or anæmia after accidents or surgical operations. The pregnant woman usually suffers from a disturbance of general bodily metabolism, as shown by alterations in her blood and urine. The general tendency in the later months of pregnancy is towards constipation of the bowels, while there may be at the same time slight albuminuria or glycosuria. It is probable that an accumulation of nitrogenous metabolic products belonging to the xanthin and uric-acid series also takes place in the blood, which is itself hydræmic at this period. Some of these conditions are transient, but others are more persistent, and tend to act on the brain and body generally so as to lessen the patient's powers of resistance to agencies which produce bodily disease or cerebral disorder. According to Hansen and Idanof,[10] from 70 to 80 per cent. of the puerperal psychoses arise from septic infection or toxic agencies, while in the remaining 20 to 30 per cent. of cases such ætiological factors were absent or could not be traced. As regards the clinical forms of puerperal insanity, Hoppe[11] found that out of 100 cases, 63 showed hallucinatory delirium and acute mental confusion, 11 exhibited melancholia, 11 were of the type of periodic insanity, seven showed mental disturbance with marked hysteria, five were markedly hallucinatory, while one was epileptic, and two showed signs of general paralysis. Next to hallucinatory delirium with mental confusion, melancholia is the most frequent clinical form of the puerperal psychoses. The various exciting causes, such as toxæmia and anæmia, anxiety and fear, pain and exhaustion, and in some cases the shame of illegitimacy, may, especially in primiparæ, co-operate with a varying element of predisposition, so as to give rise to one or other of the various manifestations of puerperal insanity. Epilepsy and general paralysis may be present as complications, and careful inquiry will then show the presence of these in the history of the patient prior to pregnancy or the puerperism.

[1] Boston Med. and Surg. Journ., March 21, 1901. [2] Jones, Lancet, Aug. 17, 1901. [3] Ibid. [4] Boston Med. and Surg. Journ., loc. cit. [5] Journ. of Mental Science, April 1899, p. 286. [6] Die Puerperalen und Pyæmischen Processe, Leipzig, 1873. [7] Micro-organisms and Disease. [8] Suppuration and septic diseases (New Sydenh. Soc., 1886). [9] Dent. Medic. Wochenschrift, 1895. [10] Neurolog. Centralbl., 1893. [11] Archiv. für Psychiatrie, Bd. xxv.

